# Mr. Fogo, in Answer to Dr. Trotter

**Published:** 1805-03-01

**Authors:** A. Fogo

**Affiliations:** Newcastle


### *To the Editors of the Medical and Physical Journal.*

Gentlemen,

THE publication of Cases, and animadversions upon them, have been admitted in all ages and times; it has prevented the ignorant and illiberal mind from taking shelter under the recovery of the patient, as daily done by the Quack and the Charlatan; it has given spirit to discussion; and collisions of sentiments and opinions, have in a great measure brought the healing art to its present perfection.*

In No. 68 of the Med. and Phys. Journal, Dr. Trotter earnestly requested medical men to offer their *animadversions* on a case he then published. As no notice has been taken of it in the Journal; and as Dr. T. has sent another paper on the same subject in No. 72, and promises a third, I am inclined, with your permission, to make a few animadversions on the case. Non tantas componere lites, between two such eminent physicians, but to mention some doubts I entertained on my first reading the case, respecting the nature of it. As Dr. Trotter has solicited the medical world to speak out on the occasion, I hope he will not take offence at what I have written, as he may be well assured, no offence is intended.

Dr. Trotter is pleased to call the disease *Gastrodynia.* Among the most eminent systematic nosological writers, that word is used as a generic term by Sauvages only. He has mentioned twenty different species of it, from different and opposite causes; all which Dr. Cullen has included among his species of the genus Dyspepsia. Sauvagesii definitio: Quicumque dolor notabilis et constans in regione stomachi, qui continua animi defectione non stipatur ut cardialgia, nec pyrexia ut gastritis. Or. IV. g. XXI.

Dr.

* Dr. Trotter's Letter, Medical and Physical Journal, No. 72.

Dr. Trotter describes the symptoms of the disease, and says, there was "a most acute pain in the epigastric region towards the right hypochondrium, under the last rib, and darting from thence acutely upwards to the shoulder of the same side; pulse 140, and moderate."

These symptoms appeared to me at first sight to belong to a very different and more dangerous disease; and the result of the case, I hope, will support that opinion.

As there is nothing similar in the definitions given by Sauvages and Dr. T. it may be presumed the disease was not Gastrodynia; but as my opinion can have little or no weight in the estimation of Dr. T. I will call to my assistance the opinion of our venerable preceptor. His definition of hepatitis is, (Cullen Synops. g. xvii.)

"Pyrexia, tension and pain in the right hypochondrium; sometimes pungent like that of pleurisy, but more frequently obtuse; a pain reaching to the clavicle and top of the right shoulder; a difficulty of lying on the left side; dyspnœa; dry cough, vomiting, and hiccup."

These symptoms are so very synonymous with those Dr. Trotter has given, that, I hope, if he will dispassionately compare them, he will be inclined to renounce, at least to moderate, his opinion.

In the definitions of the inflammations of all the other abdominal viscera, there is not a word said of "a most acute pain in the epigastric region towards the right hypochondrium, under the last rib, and darting from thence upwards to the shoulder of the same side." These are the unequivocal symptoms of hepatitis.

I will not trouble you with the daily reports, but refer you to the case at large, No. 68 of your Journal. I however beg to call your attention to the minutes of what passed on the seventeenth day.

"A purging mixture was ordered last night. She had six motions in the course of this day, resembling coffee-grounds, without any mixture of fæcal matter."

I wish to know, from whence came so great a quantity of coffee-coloured matter? From the frequent involuntary discharge upwards by vomiting, and downwards by purgatives, few medical men will be hardy enough to prove it was lodged in the stomach or bowels. Is it possible that these six evacuations of coffee-coloured matter could come from any quarter so likely as from the liver, which had been in a state of inflammatory obstruction during the illness? It seemed a critical termination of the disease. And behold, "On the nineteenth had a good night, pulse 98." The pulse fell from 140 to 98; and during Dr. T's attendance, it never exceeded 114 in the evening.

